# A father’s crusade in rare disease drug development: a case study of Elpida therapeutics and *Melpida*

**DOI:** 10.1186/s13023-025-03892-0

**Published:** 2025-07-16

**Authors:** Deanna Portero, Qingyang Xu, Aaliya Hussain, Andrew W. Lo

**Affiliations:** 1 Johns Hopkins Bloomberg School of Public Health, Baltimore, MD USA; 2 Johns Hopkins Carey Business School, Baltimore, MD USA; 3 MIT Laboratory for Financial Engineering, Cambridge, MA USA; 4 MIT Sloan School of Management, Cambridge, MA USA; 5 MIT Computer Science and Artificial Intelligence Laboratory, Cambridge, MA USA; 6 Santa Fe Institute, Santa Fe, NM USA

## Abstract

Therapeutic development for rare diseases is difficult for pharmaceutical companies due to significant scientific challenges, extensive costs, and low financial returns. It is increasingly common for caregivers and patient advocacy groups to partner with biomedical professionals to finance and develop treatments for rare diseases. This case study illustrates the story of Terry Pirovolakis, a father who partnered with biomedical professionals to develop the novel gene therapy, Melpida, within 36 months of the diagnosis of his infant son. We identify the factors that led to the success of Melpida and analyze the business model of Elpida Therapeutics, a social purpose corporation founded by Pirovolakis to reproduce the success of Melpida for other rare diseases. We conclude with four lessons from Melpida to inform caregivers like Pirovolakis on developing novel gene therapies to save their loved ones.

## Introduction

In mid-2018, Terry Pirovolakis’s wife Georgia, noticed that their 5-month-old son Michael missed important development milestones: Michael exhibited signs of intellectual disability, his motions were floppy (hypotonia), and the size of his head was in the lowest 10th percentile of children at his age (microcephaly).

Initially the Pirovolakises were not concerned as they believed that Michael would soon catch up in his growth. One month later, the symptoms persisted and they visited their family doctor, who suspected that Michael was infected with Zika or cytomegalovirus due to Mr. Pirovolakis’s recent work trips to Latin America. To their great relief, the infectious disease tests were negative, but they still had no diagnosis. Anxious and with no prior background in biomedicine, Mr. Pirovolakis—a 38-year-old Canadian IT professional—delved into the biomedical literature to understand his son’s symptoms and search for answers. When an MRI scan revealed abnormal white matter in Michael’s brain and Michael suffered a major seizure soon afterwards, Michael’s neurologist decided to perform whole exome sequencing in the hope of finding a diagnosis.

On April 2, 2019, the Pirovolakis family received devastating news: their now 15-month-old son suffered from Spastic Paraplegia Type 50 (SPG50), a severe form of complex Hereditary Spastic Paraplegia that initially presents with hypotonia, microencephaly, delayed development, and febrile seizures, eventually progressing to debilitating lower extremity spasticity and weakness, moderate upper extremity spasticity and weakness, moderate-to-severe intellectual disability, and behavioral issues [1, 2]. SPG50 is an ultra-rare genetic disease that was known to affect fewer than 100 people worldwide and only about 16 patients in North America [2]. Worse still, there were no available therapies to treat Michael and his life expectancy was largely unknown.

After two days in anger and agony, the Pirovolakises resolved to act, making swift progress due to a confluence of determination, resourcefulness, and means. Within 48 hours of receiving the diagnosis, they founded a charity named CureSPG50 for fundraising and discussed drug development strategy with the leaders of Cure SPG47 (a genetic disease closely related to SPG50). They created an initial pool of research funding capital by refinancing their home.

At the same time, Mr. Pirovolakis quickly established a network of mentors and contacts in the biopharmaceutical industry, demonstrating a remarkable social ability to connect and garner the support of senior, elite biomedical stakholders. He sought advice on therapeutic development from the founder and CEO of a publicly traded biotech coempany developing treatments for rare and ultra-rare diseases. The CEO invited Pirovolakis to participate in a non-profit hosted training program connecting biomedical experts in the rare disease field to caregivers like Pirovolakis, who are interested in developing rare disease treatments to save their loved ones [3]. Pirovolakis also discussed creating preclinical mouse models with the Vice President of a preclinical biomedical research institution, and received support to conduct a clinical trial on Michael from the CEO of a pediatric hospital in Canada.

In the following weeks, Pirovolakis attended several prominent biomedical conferences. During these events, he engaged with leading figures in the biotechnology sector and concluded that gene therapy was the optimal approach for treating Michael, as antisense oligonucleotides (ASOs) technology was not amenable to his son’s disease. In particular, Pirovolakis connected with a leading gene therapy researcher who served as director of a translational gene therapy core at an academic medical center. The researcher agreed to develop an adeno-associated virus (AAV) gene therapy for SPG50 and conduct a proof-of-concept (POC) preclinical study in mice [4]. Pirovolakis named the gene therapy Melpida, a combination of “Michael” and “Elpida” (the Greek word for “hope”). With a clear understanding of the financial requirements for completing a gene therapy project, Pirovolakis launched a fundraising campaign on GoFundMe. This campaign successfully raised $2.8 million, thanks to the support from his Toronto community and tens of thousands of donations from around the world and the intangible qualities of the Pirovolakis family, including their persistence and charisma.

The Pirovolakises and their team persisted for three years, surmounting significant challenges in therapeutic development during the COVID-19 pandemic. On December 30, 2021, Health Canada approved the clinical testing of Melpida in an open-label, single-arm phase I clinical trial. On March 24, 2022—just three years after his original diagnosis—Michael became the first patient to receive Melpida in Canada, and they subsequently opened a Phase I/II trial in the US where three affected children enrolled in the trial and received the treatment. Within a year of Michael’s gene therapy, he exhibited stabilization of spasticity, one of the efficacy outcomes for the trial, as measured by Tardieu and modified Ashworth scales [5]. He also exhibited positive trends across several exploratory assessments, including the Vineland Adaptive Behavior Scale, CGI of Overall Change by Physician, and Bayley Scales of Infant and Toddler Development [5].

## What enabled Melpida’s accelerated clinical timeline?

**Fig. 1:**
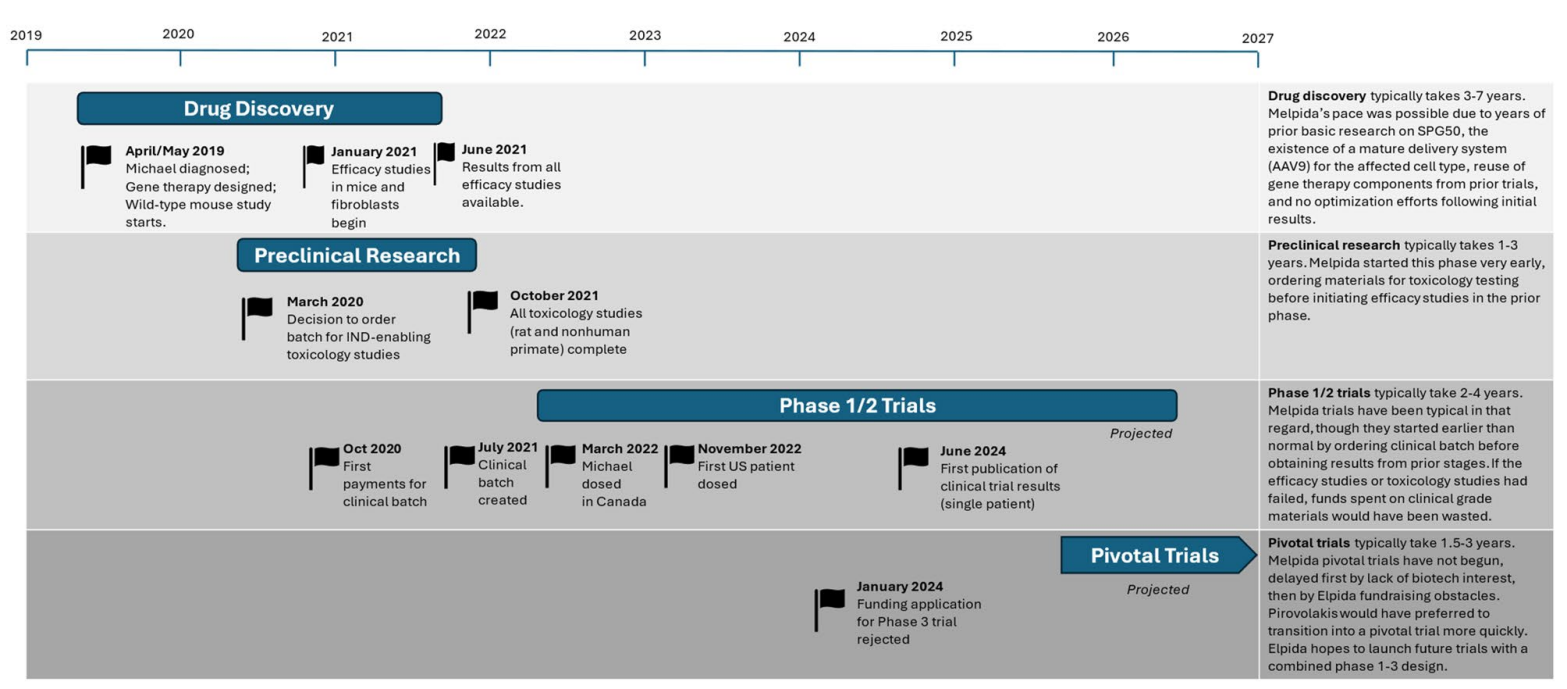
Timeline of Melpida development [6]

The gene therapy Melpida [4] was designed, tested in vivo, manufactured, and administered to Michael within 36 months of Michael’s diagnosis (Fig. 1), a miraculous achievement since Pirovolakis had no prior experience or connections in the pharmaceutical industry before Michael’s diagnosis. Several important factors during the therapeutic development of Melpida led to the accelerated timeline of its translational research.

### Substantial basic biomedical research

Prior to the therapeutic development of Melpida, the biological mechanism of AP4M1, the single gene whose mutation causes SPG50, had been relatively well-studied for over two decades [7–9]. As a result, Pirovolakis did not need to invest significant time or resources to understand the basic biology of AP4M1 during the preclinical drug development of Melpida. If prior biomedical research were less substantial, the timeline to develop Melpida would be greatly delayed.

### Mature gene therapy technology

Melpida employs the AAV serotype 9 (AAV9) as its therapeutic delivery vector. AAV9 is a widely used gene therapy delivery vector due to its ability to transduce a diverse set of cells and tissues [10, 11]. It has been used in FDA approved gene therapies such as Zolgensma (onasemnogene abeparvovec-xioi) [12] and Elevidys (delandistrogene moxeparvovec-rokl) [13]. Pirovolakis did not need to generate novel biodistribution data for Melpida regulatory submissions. Instead, he cited AAV9 biodistribution data generated by other projects that used the same route of administration. Using this mature gene therapy technology also reduced the scientific risks, as there is ample clinical evidence of its therapeutic efficacy and adverse effects. Pirovolakis leveraged the safety profile and immune management protocols from other AAV9 clinical trials that used the same route of administration to streamline and accelerate his path to the clinic.

### Experienced scientific team

Pirovolakis partnered with a team of highly qualified and compassionate clinical researchers of AAV gene therapy to develop Melpida. Pirovolakis chose to work with collaborators that shared the same sense of urgency and priority on speed. Prior to Melpida, the academic researcher working with Pirovolakis had designed multiple AAV gene therapies for rare genetic diseases [14–17]—several were also funded by parents of pediatric patients with rare genetic diseases. The expertise in AAV gene therapy vector engineering allowed the research team to efficiently reuse a promoter from previous AAV gene therapies in Melpida. The lead academic researcher and his team designed Melpida and initiated the POC study in approximately one month.

### Higher risk tolerance

Due to the urgency of Michael’s disease progression and lack of alternative treatment options, Pirovolakis was willing to tolerate a higher risk of complete financial loss in exchange for expedited development of Melpida as a potential cure. For example, the preclinical studies of Melpida did not follow the standard stage-gating procedure, where drug developers first obtain the results of certain studies prior to initiating other studies or drug manufacturing. Instead, Pirovolakis initiated toxicology and manufacturing studies prior to obtaining the results of in vivo efficacy studies. Fortunately, researchers obtained therapeutic evidence towards the end of the preclinical stage of Melpida, with the Investigational New Drug (IND) application demonstrating efficacy in both cell and animal models.

### Individual advantages

Pirovolakis’ journey might have looked very different if he had been more average in scientific acumen, financial resources, or social capital. The development of Melpida is marked by numerous examples of these advantages: Before Michael’s diagnosis, Pirovalakis had sufficient scientific literacy to search and read biomedical literature; Michael had sufficient access to skilled medical care that he received a diagnosis at 15 months of age; Pirovolakis initially leveraged his personal financial resources for funding research projects; Within one month of Michael’s diagnosis, Pirovolakis established supportive working relationships with an elite set of stakeholders, including a top academic gene therapy developer, a biotech CEO, a VP at a preclinical contract research organization, and a hospital CEO, and in subsequent months would go on to secure help and favorable collaborative arrangements from additional highly-positioned executives at a variety of biomedical research institutions. Pirovolakis’ remarkable capabilities, resourcefulness, and networking skills played a critical role in Melpida’s accelerated timeline.

### Limitation

Although the expedited preclinical timeline allowed Michael to receive the treatment within 36 months of diagnosis, the therapeutic benefits and adverse effects of Melpida were not well understood, hindering the clinical testing of Melpida on larger groups of patients. In January 2024, a major research funding agency rejected the funding application to conduct a phase III clinical trial of Melpida to treat SPG50. Although the review panel of 15 scientific members acknowledged that the clinical researchers of Melpida “have conducted an excellent preclinical program and the proposed clinical trial is as good as one could hope for”, the panel also expressed concern that “although the rationale in terms of the basic science is excellent, it is concerning that the applicants will initiate a phase III clinical trial without strong preclinical basic science. The limited information from the phase I/II trial is a weak point” [18]. Balancing the short-term medical expediency with the long-term scientific rigor of preclinical therapeutic research is an important tradeoff that needs careful consideration. Following the publication in June 2024 of data demonstrating promising efficacy in Michael’s case [5], and supported by data from the three other treated patients, Elpida plans to reapply for a grant from the same agency with evidence of promising safety and efficacy data in patients.

## From Melpida to Elpida

The total cost of therapeutic development and clinical testing of Melpida was $4.5 million, including $2 million for drug manufacturing, $1 million for safety testing, $1 million for clinical trial operations, and $0.5 million for preclinical efficacy studies.

Initially, Pirovolakis used personal savings, home equity, and personal debt to finance the therapeutic development. His fundraising campaign on GoFundMe and his charity CureSPG50 raised over $2.8 million. Pirovolakis also received approximately $3 million worth of support of in-kind donations and discounts, including donations of non-human primates for preclinical testing, substantial discounts on drug manufacturing, and a discounted fee to prepare the preclinical data of Melpida for regulatory review. A Canadian pediatric hospital agreed to treat Michael at no cost, providing services valued at $250,000.

After Michael and two other children received the treatment in 2022, Pirovolakis did not have sufficient funding to manufacture more doses of Melpida. However, the crusade to develop Melpida instilled in Pirovolakis the social responsibility to provide treatment to other patients like Michael. He contacted several pharmaceutical companies to manufacture Melpida and conduct phase III clinical trials. However, none of the companies expressed interest in bringing a one-dose treatment for an ultra-rare disease to market. Pirovolakis also applied for funding from a prominent public–private partnership initiative for gene therapy development, but was not eligible for full participation on the ground that Melpida was too advanced in the clinical trial pipeline. By late 2022, Pirovolakis concluded that his non-profit approach to drug development was not financially sustainable to provide patient access to life-saving gene therapies in the long run, and the next stage of development would require different forms of financing that were largely inaccessible by non-profit organizations: debt financing and small business grants.

Determined to save more children like Michael, Pirovolakis founded Elpida Therapeutics, a social purpose corporation (SPC) in May 2023. Launched initially with $20 million in cash and in-kind commitments, the company’s initial shareholders included individuals and organizations that played a critical role in Pirovolakis’ journey to develop Melpida. The company’s mission is to become a self-sustaining venture with an exponentially growing portfolio of transformative gene therapies from preclinical research to FDA approval [19]. Unfortunately, due to the significant downturn in the biotech industry, nearly $10 million in commitments were deprioritized as companies reduced their spending.

In addition to SPG50, Elpida’s current portfolio includes an AAV gene therapy for Charcot-Marie-Tooth disease type 4J (CMT4J), another rare genetic disorder. This therapy is in the preclinical stage and funded by a $4 million IND enabling grant from a biomedical research funding agency. The company aims to have five gene therapy candidates for rare diseases in the clinical pipeline within the next three years, with each therapy treating eight to 12 children.

At the time of this writing, Elpida had initiated a compassionate use study in Spain and the UK and was preparing Phase IIb studies in Italy and Germany to treat an additional 12 patients this year. This expansion was made possible by the efforts of foundations and industry supporters, all aiming to save more children. Elpida is also in discussions with the NIH and FDA to commence a Phase III study in 2025.

## Elpida’s approach for rare disease drug development

### Drug candidate selection

Elpida’s initial criteria for selecting new gene therapy candidates are largely inspired by the success of Melpida. Promising therapeutic candidates should (1) treat a neurodegenerative or neurodevelopmental disease, (2) have strong preclinical studies data for safety, efficacy, biodistribution, and immunology, (3) utilize AAV9-based capsid with non-systemic routes of drug administration, and (4) have sufficient data on patients’ natural history. The portfolio of gene therapies is expected to generate synergy in clinical research and further reduce the costs by sharing the same drug development platform and clinical trial infrastructure. Similarly to the funding issue, the economic downturn led to multiple rare disease programs being discontinued by biotech companies. In response, Elpida has shifted its mission to focus on rescuing late-stage programs that currently lack the means to progress to clinical trials, despite having developed the drug, filed INDs, and, in some cases, selected patients.

### Clinical trial design

Due to the rare and ultra-rare incidence of the diseases, instead of the standard clinical trial staging with three distinct phases, Elpida plans to conduct clinical trials in a single-arm study that combines all three phases in one trial. To provide patients with maximal access to potentially transformative gene therapies, the clinical trials will use natural history data as the control arm data. The goal is to generate sufficient clinical evidence for regulatory approval with as few as eight patients. This efficient trial design yields considerable cost savings in both clinical trial operations and drug manufacturing.

### Business model

Currently Elpida uses a combination of in-kind donations, research grants, loans, discounts, promissory notes, and deferred payment to finance its translational research and clinical trial programs. In the future, the company envisions that the resale of Priority Review Vouchers (PRVs) in secondary markets would become the main source of revenue.

Created in 2007, PRV is a voucher program of US FDA to incentivize therapeutic development for rare pediatric diseases, which are unfavorable to most drug developers due to the significant translation research costs and low financial returns. Once its application for Rare Pediatric Disease Designation and Biologics License Application are approved by the FDA, the drug developer may sell the PRV in a secondary market to other drug developers, who can redeem the PRV to expedite the regulatory review process for their own drug candidates. Many companies rely on the PRV resale as an important means to finance their translational research and clinical testing costs [20].

Elpida estimates that reselling a PRV to a large pharmaceutical company generates approximately $100 million in revenue in today’s market. In contrast, the total cost to develop a gene therapy in its portfolio to commercialization is about $20–$35 million. This price discrepancy allows Elpida to reinvest the profit of developing one gene therapy to fund the translational research of even more gene therapy candidates, with the hope for an exponential growth of its gene therapy portfolio. Currently the company expects to receive two PRVs for the initial five gene therapy programs, which allows it to develop 12 to 16 additional programs in the future [19].

Once a gene therapy is approved by the FDA, Elpida will out-license the drug to larger pharmaceutical companies with mature manufacturing pipeline and commercial operations. If a partnership cannot be established, Elpida will build its own commercial operations and use cost-plus pricing to ensure drug affordability and patient access to transformative gene therapies.

### Challenges

Elpida’s unconventional approach for rare disease drug development also faces its unique challenges. The most important challenge is its reliance on the resale of PRVs as the main source of revenue. In the absence of congressional action, the rare pediatric disease PRV program will fully sunset in late 2026 [21]. Under the current FDA guidelines, if a drug candidate did not receive the rare pediatric disease designation by December 20, 2024, the drug candidate will no longer be eligible for the PRV. Reauthorization bills have been introduced in Congress in 2024 and 2025, but the fate of the program remains uncertain [22]. Consequently, Elpida may need to revamp its business model to avoid significant disruptions after the sunset of PRV.

Another major challenge is to sustain the fast cash burn with its limited cash reserves and revenues in the near term. Elpida estimates that each gene therapy program will take 3 to 8 years from preclinical research to commercialization. With the growing size of its gene therapy portfolio and operations costs, it is increasingly difficult to replicate the rapid drug development timeline of Melpida and generate revenue in the near term. Therefore, Elpida’s business model is not self-sustainable and must still rely on external sources of funding.

Finally, although Elpida employs a highly qualified scientific team and a mature platform technology of AAV gene therapy, it is difficult to recruit patients in its clinical trials due to the low incidence of such rare diseases. As a result, Elpida must utilize unconventional clinical trial design with few patients, which implies greater statistical variability in clinical trial outcomes and, consequently, greater uncertainty of the regulatory review. Due to this intrinsic and unavoidable challenge of rare disease drug development, careful selection of clinical trial primary endpoints and close collaboration with regulatory agencies are critical to commercialization.

## Lessons learned

We conclude with four important lessons on rare disease drug development drawn from the remarkable journey of Pirovolakis and Melpida.

First and foremost, Melpida’s success proves that caregivers of patients with no experience in drug development can develop novel therapies to save their loved ones, if they actively engage with the biomedical community, identify the right resources, connections, and support, and never give up hope along the way. Right after learning the devastating diagnosis, Pirovolakis started to build a network of connections with biotech entrepreneurs, patient advocacy groups, and clinical researchers, who, in turn, pointed him to the financial resources, therapeutic mechanism, and clinical expertise to develop Melpida.

In addition, while Melpida’s success was inspired by love and hope, it also depended on a series of key decisions made by Pirovolakis and his team to navigate the practical issues of drug development. For instance, having an academic investigator provide the site for the clinical trial greatly reduced the trial’s cost, and connecting with higher level representatives of an organization increased the amount of donations and discounts received. A critical decision that expedited the timeline was producing the clinical grade drug before completing the toxicology studies. This choice saved the team nearly two years but carried the risk of losing all funding if the toxicology results were unsatisfactory.

It is important to receive advice from experienced mentors. For Pirovolakis, the mentorship from the biotech CEO and connections from the non-profit training provided much of the critical domain knowledge and advice. Pirovolakis now shares his learning with a digital community of more than 200 caregivers developing novel treatments for their loved ones, and this article is intended to further support other parents who find themselves in his situation.

Finally, despite the initial success of Melpida, Pirovolakis still encountered many challenges after he founded Elpida to scale AAV gene therapy development for rare diseases. The difficulty to recruit patients in clinical trials, the low financial returns generated by commercial gene therapies, the unlikely partnership with large pharmaceutical companies, and the looming potential sunset of FDA’s PRV program all pose serious challenges to the financial sustainability of Elpida. Innovations in the business models of rare disease drug development [23, 24], close partnership between public and private sectors [25], and incorporating patient values in the regulatory review process [26] are necessary to address these challenges and enable more patients like Michael to receive transformative therapies.

Pirovolakis acknowledges that Elpida’s model is not perfect or ideal, but has a more pragmatic perspective: “What other options do we have to help save children? Funding programs with fewer than 2,000 patients globally is not in the cards right now. In the end, I can either give up and return to my old career or I can move heaven and earth to try and save these children. I have never given up on anything important in my life, and I don’t intend to start now. Giving our children a better life is simply too important.”

## Data Availability

The data supporting the findings of this study consist of interviews, which were recorded in audio and video format. Due to privacy and confidentiality considerations, these recordings are not available for public sharing. Further details or transcripts may be available upon reasonable request, subject to interviewee consent and appropriate permissions.
